# Impact of sensorimotor mismatch on virtual reality sickness and user experience: age-related differences in a randomized trial

**DOI:** 10.1186/s12984-025-01677-x

**Published:** 2025-07-03

**Authors:** Elisabeth Jochmann, Thomas Jochmann, Maximilian Weber, Karolin Weigel, Carsten Klingner

**Affiliations:** 1 Department of Neurology, Jena University Hospital, Jena, Germany; 2 Department of Computer Science and Automation, Technische Universität Ilmenau, Ilmenau, Germany

**Keywords:** Proprioceptive conflict, Motion sickness, Aging, Cognitive load, Immersive technology, Head-mounted display, Neurorehabilitation, Upper-limb motor task

## Abstract

**Background:**

Virtual reality (VR) technology offers immersive and interactive experiences and is increasingly being explored for rehabilitation therapies. However, concerns about side effects such as nausea and dizziness—collectively referred to as VR sickness—are holding back clinical translation. Sensorimotor mismatches, while potentially beneficial for motor learning, may exacerbate these effects. The age groups in VR applications differ, with younger users common in gaming and older adults prevalent in rehabilitation. This study investigated whether sensorimotor mismatches in a VR-based motor task make the experience more uncomfortable and whether older adults are more affected by these mismatches.

**Methods:**

We conducted a randomized controlled trial with 104 healthy right-handed adults, including elderly participants up to 84 years old, to cover the relevant demographics for rehabilitation. Participants were divided into three intervention groups and performed a VR ball-throwing task using an Oculus Rift S head-mounted display. The groups differed in task difficulty and exposure to deliberately induced sensorimotor mismatches. The design avoided visual-vestibular conflicts typically responsible for VR sickness and instead introduced proprioceptive mismatches during hand-object interaction. VR sickness was measured using the Simulator Sickness Questionnaire (SSQ), and user experience was assessed through a self-developed questionnaire. Statistical analysis was performed using rank-transformed ANOVA, ordinal logistic regression, and Spearman’s rho with FDR correction for multiple comparisons.

**Results:**

Results indicated no significant differences in SSQ scores among the three intervention groups, suggesting that sensorimotor mismatches do not increase VR sickness. However, the Mismatch group reported higher levels of exhaustion and frustration compared to the Error-based and Errorless groups, indicating the impact of cognitive strain and task difficulty on user experience. Interestingly, younger participants reported higher (worse) SSQ scores, while older participants experienced weaker symptoms.

**Conclusions:**

VR environments with sensorimotor mismatches during hand-object interaction tasks may be feasible for rehabilitation, as they did not lead to significant discomfort in this setting. Moreover, despite concerns about age-related susceptibility to dizziness, older adults showed high tolerance to VR, supporting its potential for broader applications in rehabilitation settings. This study was reported in accordance with the CONSORT guidelines. It was registered in the German Clinical Trials Register (DRKS00034901).

**Supplementary Information:**

The online version contains supplementary material available at 10.1186/s12984-025-01677-x.

## Background

Virtual reality (VR) technology has evolved from a gaming tool into a versatile platform for education, rehabilitation, and training. VR enables immersive, interactive experiences and can replicate real-world scenarios in controlled environments, offering substantial potential to advance biomedical research and clinical interventions. VR holds considerable promise for enhancing motor learning capabilities [1, 2] and can be effectively augmented with motion tracking to provide detailed feedback and support adaptive training. One innovative approach involves deliberately inducing sensorimotor mismatches to challenge the motor system, with the aim of enhancing neuroplasticity and promoting more robust motor learning [3, 4]. However, the induced mismatches raise concerns about increasing discomfort and decreasing user engagement.

With or without deliberate sensorimotor mismatches, the adoption of VR in rehabilitation can be limited by side effects like dizziness, vertigo, and nausea [5, 6], collectively referred to as VR sickness, also termed visually induced motion sickness or cybersickness [7]. Longstanding research on motion sickness and simulator sickness has established the sensory conflict theory [8, 9], suggesting that sickness arises when there is a novel and unresolved discrepancy between expected and actual sensory input across modalities, including vision, proprioception, and vestibular signals. VR sickness shares symptom domains with simulator sickness but is not identical [6]. Disorientation symptoms, such as dizziness and vertigo, are more prominent in VR sickness, whereas the dominant symptom in simulator sickness is typically nausea [5, 6]. Conforming with the sensory conflict theory, VR sickness is commonly attributed to sensory conflicts between the user’s perceptual systems, most often involving a mismatch between visual and vestibular inputs, delayed system responses, and discrepancies in visual rendering, among other factors [7, 8, 10–12]. Foundational models of motion sickness [8, 9] acknowledge that *proprioceptive* mismatches may also induce symptoms, particularly when they are unexpected and persistent. However, empirical evidence on proprioceptive mismatch mechanisms in VR remains limited, as many VR paradigms also contain a visual-vestibular mismatch, where the visual system indicates self-motion while the vestibular system signals stasis. In the present study, the participants remained seated, and the VR scene did not include any optic flow or viewpoint movement. Thus, there was no visual-vestibular conflict—only motion of the user’s virtual arm and a ball. This design allowed us to isolate this less commonly studied conflict: mismatches between visual and proprioceptive input (sensorimotor mismatch).

Furthermore, individual differences may modulate the susceptibility to VR sickness. For example, age-related changes in multisensory integration, including alterations in visual-vestibular processing [13, 14], could influence how users adapt to sensory conflicts. While some studies have reported increased susceptibility to VR sickness and in general to dizziness and vertigo in older adults [7, 15, 16], recent systematic reviews have found that older adults typically report only mild VR-induced symptoms, suggesting no clear age-related increase in susceptibility [17–20]. Similarly, sex differences have been observed in some studies, with females potentially being more susceptible to VR sickness, although findings remain inconsistent [11, 21, 22]. These inconsistencies underline the need for further investigation, especially in the context of sensorimotor mismatches in VR.

Given these considerations, our study investigates the impact of sensorimotor mismatches on VR sickness and user experience in a VR motor task, with a specific focus on older adults. We hypothesize that older individuals, who constitute a significant demographic in rehabilitation settings, may experience greater discomfort from these mismatches, potentially limiting their engagement with VR-based therapies. Additionally, we aim to identify factors that could enhance the design and implementation of VR training programs to minimize adverse effects and improve user experience, particularly for older users. The inclusion of a substantial number of older adults, many of whom had no prior experience with computer games or VR, ensures that our findings are directly relevant to the target population for rehabilitation.

We tested the following main hypotheses:

1. *A VR motor task with artificial sensorimotor mismatches will result in greater VR sickness compared to a VR motor task without mismatches.*
2. *Older adults will experience greater VR sickness from a VR motor task, especially with artificial sensorimotor mismatches, compared to younger adults.*

Furthermore, we examined the effect of sex on VR sickness and the effect of age and sex user experience. Lastly, we analyzed factors that influence user experience in the VR session.

Through this study, we aim to contribute to the development of effective and user-friendly VR applications that minimize adverse effects while maximizing user engagement, adherence, and satisfaction—particularly for older adults in rehabilitation settings.

## Methods

### Study design

This randomized controlled trial was conducted and reported in accordance with the CONSORT 2010 guidelines. It used a parallel design with 104 healthy right-handed adults allocated in a 1:1:1 ratio to one of three intervention groups: Mismatch, Error-based, and Errorless. Demographic data, including further information like daily time spent at a PC and prior VR experience, were collected using a questionnaire before the intervention. Participants then underwent a motor task in a VR environment (‘VR motor task’). Following the VR task, VR sickness and user experience were assessed with questionnaires.

### Participants

We recruited 104 healthy, right-handed participants (62 females, 42 males, 19–84 years old, mean age 50.0 ± 21.7 years, median age 59.0 years) from a larger study investigating the effects of sensorimotor mismatch on motor learning. The sample size was determined based on an *a priori* power analysis for the primary outcome of that original study: motor learning performance across three groups. The same participant cohort was used for the present study.

The power analysis was performed using a repeated-measures ANOVA framework to detect between-group differences, assuming a medium effect size (f = 0.35) as defined by Cohen (1988), a significance level of α = 0.05, and a power of 80% (β = 0.20) [23]. The resulting required sample size was 84 participants (28 per group). To account for potential attrition—particularly due to motion sickness during VR exposure—we increased the recruitment target by 20%.

Inclusion criteria required participants to be 18 years or older, capable of providing consent, and right-handed. Right-handedness was ascertained using the Edinburgh Handedness Inventory [24]. Exclusion criteria included pre-existing functional impairment of the right upper extremity, severe visual impairment, and known neurological or psychiatric disorders.

The study took place in a research building of Jena University hospital in Jena, Germany between April 2021 and July 2022. Participants were compensated for their involvement. Written and personal explanations of the trial's procedures were provided, and written consent was obtained following the Declaration of Helsinki II. The ethics committee of the Medical Faculty at Friedrich Schiller University Jena, Germany, approved the trial (registration number: 2019-1447-BO).

Participants were randomized centrally using an online randomization tool operated by the Jena Center for Clinical Studies. This process was conducted prior to the initiation of the study for each participant and was entirely independent of the principal investigator, ensuring that randomization could not be influenced. An independent statistician generated the randomization list using nQuery Advisor 7.0, employing a deterministic pseudo-random number generator. This method ensures reproducibility for the same seed. Randomization was blocked with mixed block lengths of 3 and 6, without stratification. The enrolling investigator allocated the participants via an internet tool (PaRANDis) in the order of appearance for the enrollment visit. The investigator had no prior access to the randomization list or seed. Participants were aware that the study involved different task conditions, potentially including sensorimotor mismatches, but were not informed of their specific group assignment.

### Virtual reality motor task (intervention)

The VR motor task was conducted using a head mounted display (Oculus Rift S virtual reality headset, Meta Platforms, Inc., single fast-switch LCD panel with a per-eye resolution of 1280 × 1440 pixels and a refresh rate of 80 Hz) with the bundled right-hand controller, connected to a laptop (HP Omen 15-dh0010ng). We used the motion tracking feature from the VR headset, which tracks six degrees of freedom with inside-out tracking using five built-in cameras [25]. Our custom-developed VR software, created using Unity (Version 2019.4.7f1), is publicly available [26].

**Fig. 1:**
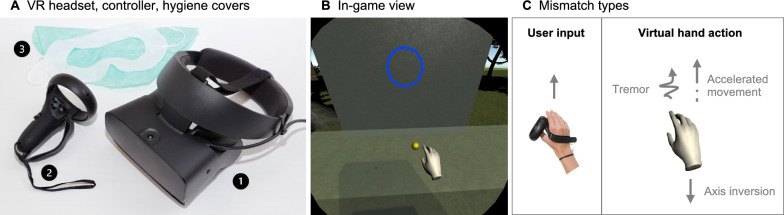
VR hardware, software interface, and sensorimotor mismatch types. **A** Oculus Rift S headset (1), right-hand controller (2), disposable VR cover (3), and hair cap (3) used during the study. **B** Screenshot of the VR software interface, showing the virtual hand, yellow ball, and blue circular target. **C** Representation of sensorimotor mismatch types, where real hand movements (user input) are altered by varying axis inversions, overlaid tremors, and amplitude changes in the virtual hand's actions

Participants engaged in a ball-throwing task within a virtual reality environment while seated, using their right hand. The pupillary distance of each participant was measured and adjusted in the Oculus software settings for optimal visual quality. The right-hand controller was secured against dropping with an additional hand strap (Kiwi Design Oculus Quest Controller Strap), encouraging realistic throwing movements. The head-mounted display was fitted securely, and disposable VR covers and hair caps were used to maintain hygiene (Fig. 1A).

Through the VR headset, participants viewed their virtual right hand in a 360° virtual environment. Within that virtual environment, a yellow ball, approximately the size of a ping pong ball, was placed on a stand within arm's reach. Participants were to pick up the ball and throw it at a bright blue circular target (Fig. 1B). Successful hits were indicated by a green circle and misses by a red circle where the ball hit. After a 3-s pause, the next ball appeared on the stand for subsequent throws (see videos of the VR ball-throwing task in the Error-based (Additional file 1) and Mismatch (Additional file 2) modes).

All participants familiarized themselves with the virtual environment and task mechanics using five balls in the Errorless learning mode. They then accomplished the respective task for their assigned group for 10 min.

Mismatch Group: In the mismatch group, a 20% hit rate was intended, with the target size dynamically adjusting to maintain this low success rate. Mismatch interventions included inversion of the x, y, and z axes of displayed hand movements, introduction of a tremor, and alterations in the displayed hand movement amplitude (Fig. 1C). These parameters were randomly combined and introduced anew for each throw to continuously challenge sensorimotor integration. The intervention was designed to promote a neural state favorable for enhanced motor learning, consistent with principles of error-based learning [27]. Further details of the sensorimotor mismatches are provided in Appendix A.

Error-based Group: In the error-based group, a 70% hit rate was intended, with the target size dynamically adjusting to maintain this success rate. Displayed hand movements were unaltered, providing a consistent sensorimotor experience.

Errorless Group: In the errorless group, a high success rate was ensured through a very large, fixed, and easily hit target. Displayed hand movements were unaltered, providing a consistent and unchallenging sensorimotor experience.

### Simulator sickness questionnaire (SSQ)

We used the SSQ [28] as an established method for quantifying sickness that has been broadly used to examine side effects of virtual reality [8, 29–31]. It consists of 16 individual items, namely general discomfort, fatigue, headache, eyestrain, difficulty focusing, increased salivation, sweating, nausea, difficulty concentrating, fullness of head, blurred vision, dizzy (eyes open), dizzy (eyes closed), vertigo, stomach awareness, and burping. The items are rated by the participants on a scale from 0 to 3 (none, slight, moderate, severe). For analysis, the items are allocated to three independent symptom clusters, Nausea (N), Oculomotor (O), and Disorientation (D), from which, a Total Score (TS) is calculated. Higher scores represent higher levels of sickness. SSQ scores were calculated according to the original methodology outlined by Kennedy et al. [28], using the corrected notation for the Total Score (TS = **(**[N] + [O] + [D]**)** × 3.74) by Bimberg et al. [29]. In our study, we used a German translation of the SSQ [32]. The SSQ was assessed immediately following the VR intervention.

### User experience questionnaire

After performing the VR motor task and filling in the SSQ, participants rated their user experience using a custom questionnaire consisting of 15 questions. The items were rated on a 4-point Likert scale from 0 to 3 (0-not at all, 1-somewhat, 2-moderate, 3-very). The questionnaire was presented to the participants in German. English translations of the items and their abbreviations, as used in the figures, are shown in Table 1.

**Table 1:** User Experience Questionnaire items and their abbreviations as used in this report

Questionnaire item	Abbreviation
The VR game was fun	Fun
The VR game was exhausting	Exhausting
The VR game was frustrating	Frustrating
The VR game was easy to play	Easy
The VR game was boring	Boring
The VR game is technically mature	Technically mature
The VR game was motivating	Motivating
Missed throws were caused by me	Missed throws caused by me
Missed throws were caused by the technology	Missed throws caused by technology
The VR headset was comfortable to wear	Headset comfortable
The image of the VR headset was sharp	Sharp image
The hand recognition was good	Good hand recognition
I would like to play again	Play again
VR is a potential method for regular training/rehabilitation	Method for rehabilitation
I am well-versed with computers and computer games	Well-versed with computers

To assess the internal consistency of the questionnaire, we conducted a reliability analysis. Three items (*"I am well-versed with computers and computer games"*, *"Missed throws were caused by me"*, and *"Missed throws were caused by the technology"* were excluded from this analysis, as they captured prior computer experience or error attribution rather than core aspects of user experience. Internal consistency for the remaining 12 items was good (Cronbach’s α = 0.80), supporting the coherence of the scale.

### Outcomes

Primary outcome was VR sickness, assessed using the Total Score of the SSQ. This outcome was used to evaluate the effects of the intervention (Mismatch, Error-based, Errorless) and of participant age, in line with the study’s main hypotheses.

Secondary outcomes included the three SSQ subscores—Nausea, Oculomotor, and Disorientation—to provide a more detailed symptom profile. We also explored the effect of sex on SSQ scores. Additional secondary outcomes addressed user experience. Specifically, we analyzed responses to the 15 individual items of the User Experience Questionnaire across intervention groups and in relation to age and sex. Furthermore, we examined associations between individual user experience ratings and VR sickness (TS of the SSQ), as well as participants’ perceptions of VR as a potential method for rehabilitation.

### Statistical analysis

SSQ scores were heavily skewed, violating assumptions of standard parametric analyses. To address this, we conducted a rank-transformed ANOVA. Specifically, each SSQ subscore was aligned by subtracting the mean of each intervention group × sex combination and adding the overall mean. The aligned data were then ranked globally, and linear regression models were fitted to these ranked outcomes. The predictors included intervention group (three levels: mismatch, error-based, errorless), sex (male, female), their interaction, and age as a continuous covariate. Effect sizes were reported as partial eta squared (η^2^ₚ), quantifying the proportion of variance in the ranked outcome attributable to each predictor.

We analyzed User Experience Questionnaire items using an ordinal logistic regression model. Each Likert-scale question was fitted separately using the intervention group (three levels) as the primary factor and including age and sex as covariates. After fitting each model, we performed pairwise group comparisons (e.g., Mismatch group vs. Error-based group) via Wald tests on the relevant regression coefficients.

Correlations between variables were analyzed using Spearman’s rho.

To control for Type I error inflation due to multiple comparisons, we applied a false discovery rate (FDR) correction to all *p*-values [33].

Statistical significance was defined as *p* < 0.05 after FDR correction.

Data were analyzed using statistical software JASP (Version 0.18.3) [34] and Python with the scipy, scikit-learn, and statsmodels libraries [35–37]. Data were visualized using Python with the seaborn library [38].

## Results

### Participant characteristics

A total of 104 adult participants (62 females, 42 males) aged 19 to 84 years (mean 50.0 ± 21.7 years, median 59.0 years) were included in the study between April 2021 and July 2022. Participants were randomly assigned to one of three intervention groups: Mismatch (n = 34), Error-based (n = 35), and Errorless (n = 35). There were no losses or exclusions after randomization and all participants were included in the outcome analysis (the CONSORT flow diagram is included in Appendix B, Fig. 5). The study ended after completion of the recruitment goal. Between the groups, there were no significant differences in age (ANOVA), sex distribution (Chi-Square), VR experience (Kruskal–Wallis, 1 item missing), or daily time spent at a PC (Kruskal–Wallis, 1 item missing) (see Table 2 for group-wise demographical statistics and Fig. 6 in Appendix C for group-wise age distributions).

**Table 2:** Participant demographics in the three intervention groups

	Mismatch	Error-based	Errorless
**Participants**			
Total	34	35	35
Female	21 (61.8%)	24 (68.6%)	17 (48.6%)
Male	13 (38.2%)	11 (31.4%)	18 (51.4%)
**Age**			
Median	61.5	62	57
Mean	51.35	49.43	49.11
Std. Deviation	20.86	22.55	22.28
Range	57	63	63
Minimum	20	21	19
Maximum	77	84	82

### Effects of intervention group, age, and sex on SSQ outcomes

None of the participants terminated the VR session prematurely for any kind of side effects including sickness. The median of the SSQ total score was 11.2 (SD 16.7), a higher score indicating higher levels of sickness. For 26% of the participants, the TS was 0; 46% scored between 0 and 20; 28% scored higher than 20.

Statistical analysis of SSQ scores revealed no significant main effects of intervention group, sex, or group × sex interaction for any of the SSQ outcomes—TS, N, O, and D (see Table 5 in Appendix D for descriptive data stratified by sex). In contrast, age showed a significant negative effect on TS (*p* = 0.016) and O (*p* = 0.016) subscores, but not on N (*p* = 0.476) and D (*p* = 0.059) subscores. These effects indicate that younger participants reported higher VR sickness symptoms in multiple domains.

**Fig. 2:**
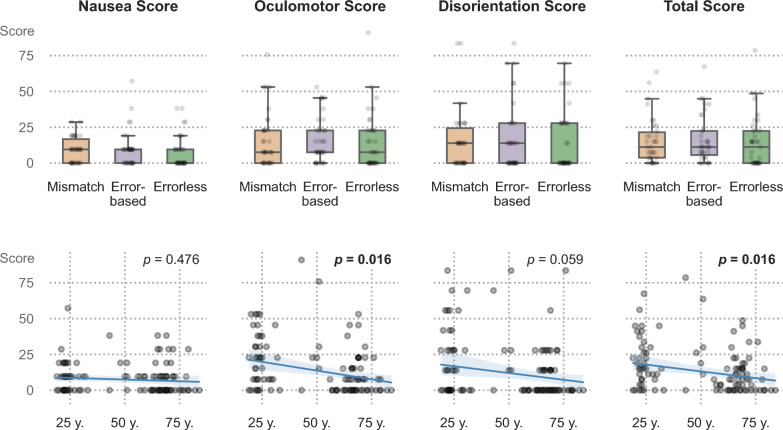
Group differences and age effects on Simulator Sickness Questionnaire (SSQ) scores. The top row visualizes the distribution of SSQ scores across the three VR intervention groups (Mismatch, Error-Based, and Errorless), with no significant differences observed between the groups. The bottom row shows regression plots with 95% confidence intervals, illustrating the relationship between age and SSQ scores. Significant negative associations were found for the Oculomotor Score and Total Score. (n = 104)

Effect sizes, *p*-values, and F-statistics for all predictors and outcomes are summarized in Table 3. Partial eta squared values showed medium effects for age on TS (η^2^ₚ = 0.093), O (η^2^ₚ = 0.123), and D (η^2^ₚ = 0.066), while all other predictors showed only small or negligible effects. Group differences and age effects are visualized in Fig. 2.

**Table 3:** ANOVA results for rank-transformed SSQ outcomes (n = 104)

Outcome	Predictor	F (df1, df2)	raw* p*-value	FDR-corrected *p*	Partial η^2^ₚ
TS	Group	0.45 (2, 97)	0.637	0.849	0.009
	Sex	3.72 (1, 97)	0.057	0.182	0.037
	Group × Sex	0.10 (2, 97)	0.909	0.919	0.002
	**Age**	**9.99 (1, 97)**	**0.002**	**0.016**	**0.093**
N	Group	0.88 (2, 97)	0.418	0.743	0.018
	Sex	1.96 (1, 97)	0.165	0.430	0.02
	Group × Sex	0.57 (2, 97)	0.569	0.828	0.012
	Age	1.41 (1, 97)	0.238	0.476	0.014
O	Group	0.71 (2, 97)	0.493	0.789	0.014
	Sex	3.81 (1, 97)	0.054	0.182	0.038
	Group × Sex	0.26 (2, 97)	0.772	0.882	0.005
	**Age**	**13.61 (1, 97)**	** < 0.001**	**0.016**	**0.123**
D	Group	0.08 (2, 97)	0.919	0.919	0.002
	Sex	1.76 (1, 97)	0.188	0.430	0.018
	Group × Sex	0.37 (2, 97)	0.693	0.853	0.008
	Age	6.80 (1, 97)	0.011	0.059	0.066

*p*-values are based on linear models applied to rank-transformed SSQ scores. F refers to the ANOVA test statistic; df₁ and df₂ denote the degrees of freedom for the effect and the residual error, respectively. False discovery rate (FDR) correction was applied across all predictors to control for multiple comparisons. Statistically significant effects are highlighted in bold. Partial eta squared (η^2^ₚ) indicates the proportion of variance explained by each predictor, controlling for other effects. According to Cohen (1988), η^2^ₚ ≈ 0.01 is small, ≈ 0.06 medium, and ≥ 0.14 large

### User experience

Amongst the responses from the n = 104 participants, there were four missing values in the User Experience Questionnaire: ‘easy’ in the Mismatch group for one participant, ‘technically mature’ in the Mismatch group for one participant, ‘play again’ in the Mismatch group for one participant, and ‘method for rehabilitation’ in the Error-based group for one participant. Missing values were omitted, leaving n = 103 responses for these four items.

Overall, the VR system and the motor task software were positively rated. The responses for the individual items for the whole cohort are depicted in Fig. 7 in Appendix E.

**Fig. 3:**
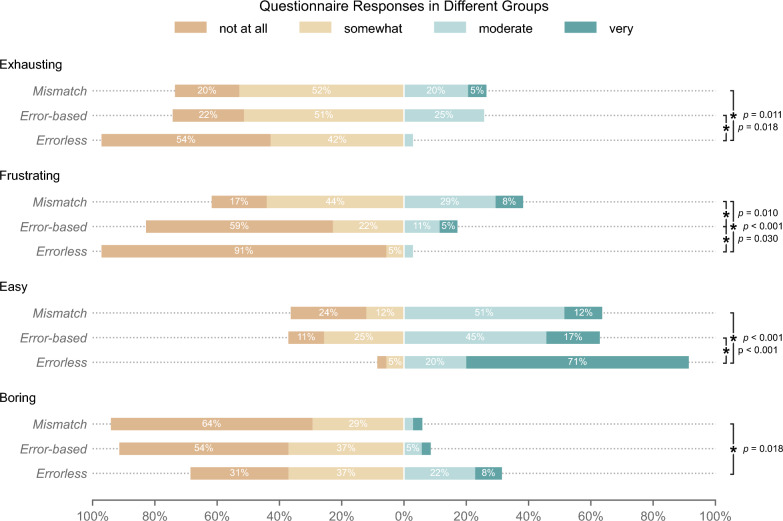
Comparison of the intervention groups' ratings on exhaustion, frustration, ease, and boredom from the User Experience Questionnaire. The chart shows the percentage of responses on a 4-point Likert scale. When comparing the three VR groups, there was a significant difference in the participants' ratings of how exhausting the VR session was, with a higher rating of exhaustion in the Mismatch vs. the Errorless group and the Error-based vs. the Errorless group. Participants' ratings of frustration differed significantly among the three intervention groups, with a higher rating of frustration in the Mismatch vs. the Error-based, the Mismatch vs. the Errorless group, and the Error-based vs. the Errorless group. Furthermore, there was a significant difference in the participants' ratings of how easy to perform they perceived the VR task in between the three intervention groups, with the Errorless group rating the task as significantly easier compared to both the Error-based and Mismatch groups. The Errorless group rated the session as more boring than the Mismatch group. The item ‘easy’ had one missing value in the mismatch group, which was omitted. This resulted in a sample size of n = 103 for the item ‘easy’ and n = 104 for all other items

Ordinal logistic regression analyses with group, age, and sex as predictors revealed significant group differences for several questionnaire items, summarized in Fig. 3.

The Errorless group rated the session as significantly less exhausting than both the Mismatch (*p* = 0.011) and Error-based groups (*p* = 0.018). Similarly, frustration was rated lower in the Errorless group compared to Mismatch (*p* < 0.001) and Error-based (*p* = 0.03) as well as in the Mismatch group compared to the Error-based group (*p* = 0.01). The Errorless group also found the task easier than both other groups (both *p* < 0.001). Further, the Errorless group rated the task significantly more boring than the Mismatch group (*p* = 0.018).

No significant group differences were found for the remaining items after FDR correction (*fun, technically mature, motivating, missed throws caused by me, missed throws caused by technology, headset comfortable, sharp image, good hand recognition, play again, method for rehabilitation, well-versed with computers*), notably including the items *how strongly errors were caused by themselves, how strongly errors were caused by the technology*, and whether *VR training could be a method for rehabilitation*.

Age showed significant associations with several User Experience Questionnaire items. Specifically, younger participants rated the task as *easier* (*p* = 0.036), were more likely to describe the task as *boring* (*p* = 0.036) and more likely to want to *play again* (*p* = 0.018). Furthermore, they indicated higher computer familiarity (*well-versed with computers*, *p* = 0.042). Sex showed no significant effects on User Experience Questionnaire items.

A detailed summary of all model results and comparisons is provided in Table 4.

**Table 4:** Ordinal logistic regression results of User Experience Questionnaire items with coefficient (c), odds ratio (OR), and FDR-corrected *p*-value for each pairwise group comparison, as well as for age and sex

Question	Mismatch vs. Error-based			Mismatch vs. Errorless			Error-based vs. Errorless			Age			Sex		
	c	OR	*p*	c	OR	*p*	c	OR	*p*	c	OR	*p*	c	OR	*p*
Fun	0.82	2.26	0.22	−0.07	0.94	0.91	0.88	2.42	0.18	0.00	1.00	0.89	−0.43	0.65	0.56
Exhausting	−0.13	0.88	0.91	−**1.60**	**0.20**	**0.011**	**1.47**	**4.35**	**0.018**	0.01	1.01	0.85	0.45	1.56	0.56
Frustrating	−**1.62**	**0.20**	**0.010**	−**3.58**	**0.03**	** < 0.001**	**1.96**	**7.07**	**0.030**	0.00	1.00	0.89	0.47	1.60	0.56
Easy	0.39	1.47	0.82	**2.80**	**16.36**	** < 0.001**	−**2.41**	**0.09**	** < 0.001**	−**0.02**	**0.98**	**0.036**	−0.95	0.39	0.28
Boring	0.36	1.43	0.87	**1.48**	**4.41**	**0.018**	−1.12	0.32	0.08	−**0.02**	**0.98**	**0.036**	−0.26	0.77	0.72
Technically mature	0.10	1.10	0.91	0.26	1.30	0.87	−0.16	0.85	0.91	0.00	1.00	0.89	−0.08	0.93	0.88
Motivating	0.66	1.94	0.32	−0.25	0.78	0.87	0.92	2.50	0.15	0.01	1.01	0.31	−0.62	0.54	0.37
Missed throws caused by me	0.05	1.05	0.91	−0.25	0.78	0.87	0.30	1.36	0.87	0.00	1.00	0.89	0.55	1.73	0.46
Missed throws caused by tech	−0.28	0.76	0.87	−1.15	0.32	0.08	0.87	2.39	0.21	0.00	1.00	0.89	0.06	1.06	0.88
Headset comfortable	−0.80	0.45	0.22	−0.07	0.93	0.91	−0.73	0.48	0.28	0.00	1.00	0.89	−0.28	0.76	0.71
Sharp image	−0.39	0.68	0.87	−0.14	0.87	0.91	−0.24	0.78	0.91	**0.03**	**1.03**	**0.036**	−0.07	0.94	0.88
Good hand recognition	0.11	1.11	0.91	1.60	4.94	0.06	−1.49	0.23	0.08	0.00	1.00	0.89	−0.14	0.87	0.88
Play again	−0.15	0.86	0.91	−0.29	0.75	0.87	0.14	1.15	0.91	−**0.03**	**0.97**	**0.018**	−0.76	0.47	0.28
Method for rehabilitation	0.41	1.51	0.82	0.55	1.74	0.55	−0.14	0.87	0.91	−0.01	0.99	0.31	−0.31	0.73	0.71
Well-versed with computers	−0.19	0.82	0.91	−0.12	0.89	0.91	−0.07	0.93	0.91	−**0.02**	**0.98**	**0.042**	−0.73	0.48	0.28

Statistically significant effects (*p* < 0.05) are highlighted in bold font

### Correlation analysis of questionnaire items

There was a significant correlation between the TS of the SSQ and the following items of the User Experience Questionnaire: the item *comfort wearing the head-mounted display* (*p* = 0.008, Spearman’s rho = −0.311, 95% CI [−0.475, −0.126]), and the item *hand recognition* (*p* = 0.001, Spearman’s rho = −0.376, 95% CI [−0.530, −0,198]).

### Fun, motivation, frustration and comfort determine the user’s evaluation of VR as a possible rehabilitation method

**Fig. 4:**
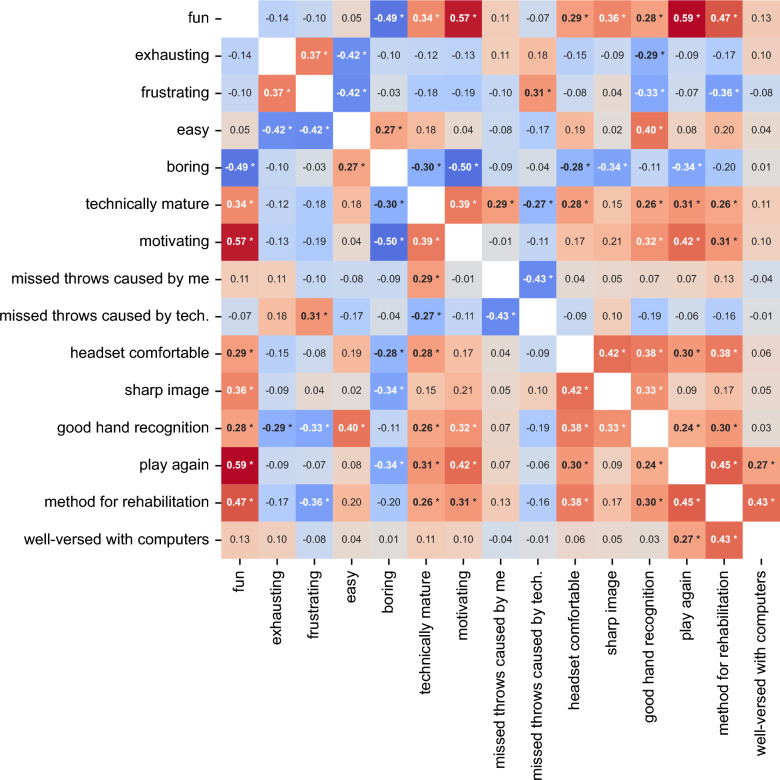
Heatmap of the correlation between answers to the User Experience Questionnaire items. This heatmap displays the Spearman's rho correlation matrix for the individual User Experience Questionnaire items. Spearman's rho measures the strength and direction of the monotonic relationship between pairs of variables, negative values indicating a negative correlation (blue), positive values a positive correlation (red). Significant correlations are indicated in bold and marked with an asterisk. Ranges for Spearman's rho effect sizes are typically interpreted as small (∣ρ∣ = 0.10 to 0.29), medium (∣ρ∣ = 0.30 to 0.49), and large (∣ρ∣ ≥ 0.50)

**Fig. 5:**
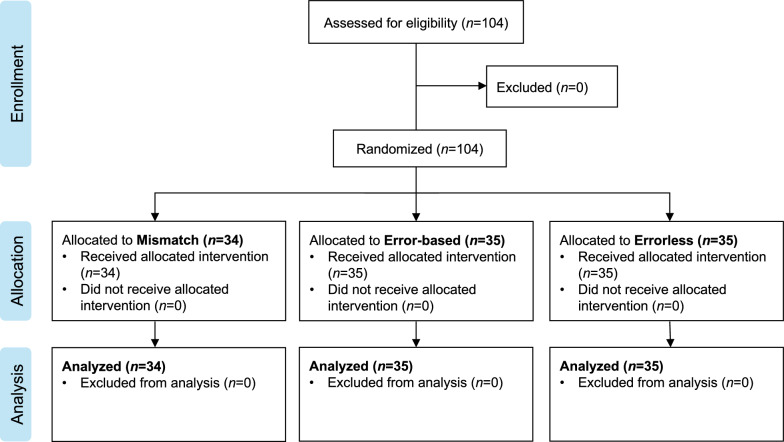
CONSORT 2010 flow diagram depicting the participant flow. The section “Follow-Up” from the original CONSORT template was omitted as the study took place on 1 day and there was no follow-up

**Fig. 6:**
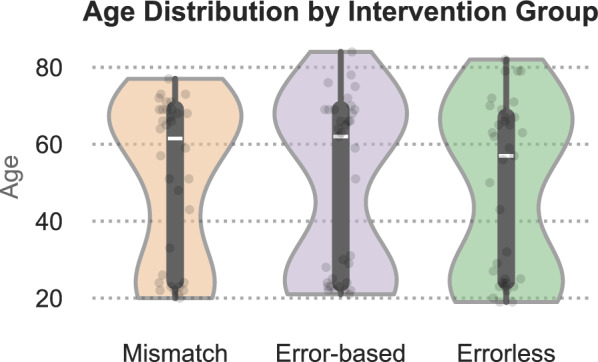
Age distribution of participants by intervention group. Each distribution includes a boxplot overlay indicating the median and interquartile range

**Fig. 7:**
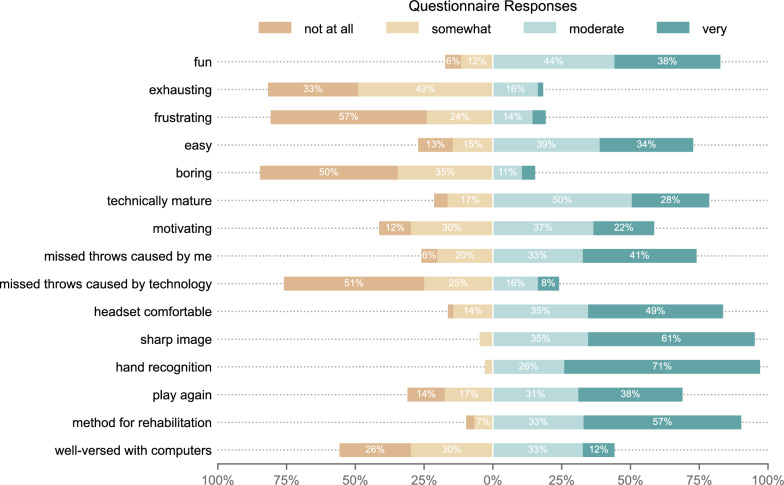
Results of individual items from the User Experience Questionnaire for all participants. The chart shows the percentage of responses on a 4-point Likert scale. Amongst the technical characteristics of the VR system, the performance of the hand recognition received the highest rating from 71% of the respondents. The sharpness of the VR display (Oculus Rift S) received the highest rating from 61% of respondents. The VR experience itself was mainly rated as fun, easy, and technically mature. Most participants rated the user experience as not exhausting, frustrating, or boring. Additionally, most participants experienced VR as a possible method for rehabilitation. Four items had missing values: ‘easy’, ‘technically mature’, ‘play again’, and ‘method for rehabilitation’, each missing from one participant. These missing values were omitted, resulting in a sample size of n = 103 for these four items and n = 104 for all other items. Table 1 shows the items in full length

Correlations between the individual items of the User Experience Questionnaire are depicted in Fig. 4. There was a significant positive correlation between the participants’ rating, if VR training could be a method for rehabilitation and their rating of fun performing the VR task (*p* < 0.001, Spearman’s rho = 0.473, 95% CI [0.307, 0.610]), their rating if the VR task was motivating (*p* = 0.007, Spearman’s rho = 0.307, 95% CI [0.120, 0.472]), their rating if wearing the head-mounted display was comfortable (*p* < 0.001, Spearman’s rho = 0.383, 95% CI [0.205, 0.537]), their rating of the hand recognition (*p* = 0.007, Spearman’s rho = 0.302, 95% CI [0.116, 0.468]), their rating if they wanted to repeat VR tasks in the future (*p* < 0.001, Spearman’s rho = 0.454, 95% CI [0.284, 0.596]), and their rating of their own experience with computers and computer games (*p* < 0.001, Spearman’s rho = 0.429, 95% CI [0.257, 0.575]). There was a significant negative correlation between the participant’s rating, if VR training could be a method for rehabilitation and their rating of the VR task being frustrating (*p* = 0.001, Spearman’s rho = −0.358, 95% CI [−0.516, −0.177]). There was no significant correlation between the participants’ TS of the SSQ and their evaluation, if virtual reality training could be a method for rehabilitation.

### Harms

No relevant adverse events or side effects were observed in any of the intervention groups throughout the study period. Participants in all groups completed the intervention without reporting relevant discomfort or adverse reactions related to the VR session. All participants tolerated the procedures well, and no participants withdrew from the study due to adverse effects.

## Discussion

### Key findings

Our study aimed to explore the acceptance and perception of VR motor tasks, with a particular focus on the impact of sensorimotor mismatches on VR sickness and user experience. Contrary to our hypothesis, sensorimotor mismatches did not significantly increase VR sickness, as evidenced by comparable SSQ scores across the Mismatch, Error-based, and Errorless groups. Additionally, older participants reported lower SSQ scores, meaning less sickness, compared to younger participants. Nevertheless, the overall acceptance of VR was positive across ages and sexes, with significant differences in how exhausting, frustrating, and easy the motor task was perceived among the different intervention groups.

### VR sickness

Despite concerns that artificial sensorimotor discrepancies might exacerbate VR sickness, our findings indicated no significant differences in the SSQ scores among the Mismatch, Error-Based, and Errorless groups. Our findings suggest that sensorimotor mismatches, in the absence of strong visual-vestibular conflicts, have a limited impact on VR sickness, indicating they are not a primary driver of sickness in VR environments [7, 8]. One possible explanation is that the mismatches introduced were not severe enough to significantly increase symptoms. Additionally, the experimental setup, where participants remained seated throughout the VR task, notably reduced the challenge to the vestibular system. This minimized vestibular input discrepancies and the likelihood of severe VR sickness. The severity of symptoms might vary with different VR tasks and setups. Furthermore, the short duration of the 10-min VR task may have contributed to the low incidence of VR sickness, as longer durations have been reported to increase the likelihood of VR sickness [21, 39–41]. Additionally, adjusting the pupillary distance in the settings of the VR software, might have helped to reduce strain on the eyes and sickness symptoms [42].

Only 28% of our participants had SSQ Total Scores above 20, a threshold that is considered indicative of significant discomfort (‘bad simulator/intervention’) [6, 28]. The majority (72%) had scores of 20 or below, highlighting that most participants tolerated the VR task well. Caserman et al. proposed adjusting this threshold for VR devices, noting a drop-out rate of about one third for SSQ scores of 40 or higher [8]. Despite 11.5% of our participants scoring higher than 40, none terminated the study prematurely. This indicates that most participants did not experience severe sickness. These findings suggest that VR environments involving hand-object interactions can include certain sensorimotor mismatches without causing substantial discomfort, which is promising for their application in rehabilitation. However, our results are specific to a seated task with limited vestibular engagement, and generalization to VR scenarios involving whole-body motion or dynamic viewpoint changes (e.g., gait training) should be made with caution.

Our correlation analysis revealed that when participants perceived the VR headset as comfortable and rated the hand recognition as good, SSQ scores were lower. The correlation between the SSQ and the comfort of the headset can be attributed to the symptoms assessed by the SSQ [43]. These symptoms directly relate to the user's comfort level while wearing the VR headset. Ergonomics and good hand recognition are crucial for minimizing simulator sickness because they ensure accurate tracking of hand movements, reducing sensory conflicts that lead to discomfort [7]. This precision enhances the sense of presence and immersion, helping to maintain user comfort and engagement by preventing VR sickness.

### Acceptance and perception of the VR motor task

While VR sickness did not significantly vary between intervention groups, the subjective experience of the VR session was markedly different. Participants in the Mismatch group and in the Error-based group reported higher exhaustion and greater frustration compared to the Errorless group. Frustration was also rated higher in the Mismatch vs. the Error-based group, confirming that sensorimotor discrepancies can challenge user experience. The increased exhaustion can be attributed to the cognitive strain induced by having to react to a new mismatch for every throw in the Mismatch group and a more challenging task in the Error-based group, unlike the Errorless group, where participants repeated the same simple task for the same duration. Furthermore, the Mismatch group had a success rate set to 20% by adjusting the target size, which explains the significantly higher frustration ratings compared to the Error-based group with a dynamic success rate of 70% and the Errorless group, which maintained a high success rate due to a very large target. Conversely, the Errorless group, designed to provide a more straightforward and less challenging experience, perceived the task as easier than the other two groups and rated the session as more boring than the Mismatch group, highlighting the balance VR applications must strike between challenge and achievability to maintain user interest, acceptance, and learning possibilities [44–47]. Moreover, perceived frustration was significantly negatively correlated with the perception of VR as a suitable method for rehabilitation, whereas exhaustion, ease, and boredom showed no significant associations.

The positive ratings for fun, ease, and perception of VR as a method for rehabilitation underscore the potential utility of VR in therapeutic contexts [48]. Our findings support that focusing on good hand recognition and visual quality, ensuring a comfortable headset, and maintaining a balance between challenge and simplicity while providing a fun and motivating environment can significantly enhance user satisfaction and acceptance [49, 50]. However, it should be noted that none of the participants had a background in or direct experience with rehabilitation, which may limit the generalizability of these acceptance ratings to clinical populations.

### Demographic influences

Our analysis revealed that older participants experienced fewer symptoms of VR sickness, aligning with a recent meta-analysis showing a lower Total Score on the SSQ for older participants [9]. However, while Saredakis et al. reported that the disorientation score was the only subscore with significantly lower values in older adults, our study found that the oculomotor score was the only subscore with significantly lower values. Overall, the literature is inconclusive about age effects on VR sickness [7, 15, 17–19, 51]. These differences in SSQ effects could be attributed to variations in the types of virtual environments used in different studies.

The age-related differences in SSQ scores could be due to variations in sensory processing [13, 14] or a lower sensitivity to sensorimotor conflicts among older adults. Lower SSQ scores in older participants are advantageous for rehabilitation, as older adults are more likely to be involved in such programs. However, the negative correlation between age and willingness to engage with VR again indicates that younger participants, despite experiencing higher sickness scores, may still be more open to using VR. Nonetheless, the acceptance of VR among older participants was still high, as shown in previous studies [19, 30, 31].

Furthermore, our study did not show significant sex differences in the SSQ, which also aligns with the meta-analysis by Saredakis et al. [9] but not with other studies [21, 22, 51]. It should be noted, however, that the overall SSQ scores were low across all groups, likely due to the limited motion stimuli in our task. This may have introduced a floor effect, potentially reducing sensitivity to detect demographic differences. These demographic insights are essential for tailoring VR interventions to different age groups, ensuring both efficacy and acceptance.

### Ratings encourage rehabilitation applications

The overall positive ratings for fun, ease, and the perceived suitability of VR as a method for rehabilitation underscore its potential utility in therapeutic settings. Notably, 89% of participants rated VR training as a suitable method for rehabilitation. Importantly, participants in the Mismatch group did not rate the item ‘possible method for rehabilitation’ significantly differently from the other groups, despite reporting higher frustration and exhaustion. While encouraging, this perception should be interpreted cautiously: none of the participants had direct rehabilitation experience or a medical condition requiring rehabilitation. Thus, their ratings reflect general impressions rather than informed assessments from a patient perspective. Nevertheless, we consider these ratings valuable as they reflect general openness to VR-based methods—particularly among older participants, who typically have less exposure to such technologies and whose acceptance was therefore less certain. Our correlation analysis further supports the role of positive user experience in perceived rehabilitation potential. Participants who rated the experience as enjoyable, comfortable, and technically robust (e.g., good hand recognition) were more likely to endorse the method as suitable for rehabilitation. Fun, motivation, and comfort—rather than an absence of VR sickness—emerged as key factors for acceptance, consistent with prior literature [52–54]. The positive correlation with prior computer experience also points to increasing acceptance in future user cohorts as digital familiarity grows. The negative correlation between frustration and acceptance for rehabilitation further underscores the need to minimize frustrating elements in VR training to enhance its therapeutic potential.

Finally, the interactive and engaging nature of VR environments can provide a stimulating context for motor training, with the potential to enhance user motivation and adherence to rehabilitation programs [1, 2]. By focusing on user satisfaction and reducing negative experiences, VR training has the potential to become a more effective and widely accepted method for rehabilitation. However, further evaluation in clinical populations will be needed to confirm these effects in therapeutic settings.

### Limitations and generalizability

Trial Limitations: While our study provides valuable insights into the impact of sensorimotor mismatches on VR sickness and user experience in VR motor tasks, several limitations should be considered. One potential source of bias is the homogeneity of the sample, as all participants were healthy, right-handed adults, which may limit the applicability of the findings to populations with different health statuses or motor impairments, who are more representative of the target group for rehabilitation. Additionally, the reliance on self-reported measures, such as the Simulator Sickness Questionnaire (SSQ) and the User Experience Questionnaire, introduces the possibility of reporting bias, as participants' responses might be influenced by their subjective perceptions rather than objective measures. The SSQ, originally developed for use in flight simulators, has not been fully validated for VR applications, which may limit its sensitivity in detecting subtle differences in VR sickness; however, it is likely sufficient to capture clinically relevant symptoms. The multiplicity of analyses, particularly in examining secondary outcomes with multiple comparisons, also raises the risk of Type I errors, despite the use of FDR correction. Moreover, the relatively short duration of the VR task (10 min) may have minimized the occurrence of VR sickness, potentially underestimating the effects of prolonged exposure. Lastly, the study's design did not account for potential carryover effects in the mismatch group, where repeated sensorimotor adjustments could have influenced both the SSQ scores and user experience over time.

Generalizability: The findings from this trial are particularly relevant to the development of VR applications for rehabilitation, given the age-diverse cohort that included a significant proportion of older adults, many of whom had limited prior experience with VR or computer games. This demographic is representative of the population most likely to benefit from VR-based rehabilitation, enhancing the external validity of the study. However, caution should be exercised when generalizing these results to populations with severe motor impairments or those undergoing long-term rehabilitation, as the trial did not include these groups. Additionally, the use of a single VR system (Oculus Rift S) and a specific motor task (ball-throwing) may limit the applicability of the findings to other VR platforms or different rehabilitation tasks. Further research is needed to explore the effects of sensorimotor mismatches in more diverse clinical populations and across a wider range of VR environments and tasks.

## Conclusion

This study suggests that VR tasks involving sensorimotor mismatches during hand-object interaction are well tolerated and positively received with minimal side effects—particularly in older adults. While discrepancies in sensorimotor feedback did not significantly increase VR sickness, they did impact user enjoyment and acceptance, underscoring that even subtle design choices can influence the user experience.

The insights into demographic influences, specifically the lower incidence of VR sickness in older participants, provide valuable direction for designing age-appropriate VR applications. Additionally, technical elements such as accurate hand recognition and high visual fidelity emerged as critical factors for enhancing user satisfaction. Addressing these elements can mitigate adverse user perceptions, making VR more accessible and enjoyable, especially in settings like rehabilitation, where user engagement is essential.

Our findings highlight the potential for VR environments to incorporate controlled sensorimotor mismatches during hand-object interaction tasks, potentially enhancing neuroplasticity and promoting motor learning without causing discomfort. Such approaches may hold promise in future rehabilitation contexts—particularly where mismatched sensorimotor feedback is inherent, such as stroke. Moving forward, optimizing VR to balance challenge and accessibility, while tailoring applications to diverse user needs, can expand VR’s impact in clinical practice, paving the way for personalized and effective rehabilitation therapies.

## Supplementary Information

Additional file 1. Video showing the Error-based training mode

Additional file 2. Video showing the Mismatch training mode

## Data Availability

The VR software used for this study is available at https://github.com/JesseRed/VRMotorLearnWurf. The datasets used and analyzed during the current study are available from the corresponding author on reasonable request and are subject to institutional review board approval.

## Appendix

### Appendix A: Quantitative details of VR-induced sensorimotor mismatch parameters in the Mismatch group

**Axis inversion**

- A random inversion of one or more axes (X, Y, or Z) was applied in each trial.
- At least one axis was always inverted in the Mismatch Group (i.e., no trials without inversion).
- The number of inverted axes was dynamically adjusted based on difficulty estimation.

**Movement amplitude changes**

- The movement amplitude was scaled randomly between 50 and 200% of the actual movement on each trial.

**Hand offset**

- The Hand position in virtual space was offset to the hand position in real space by an amount of + or −20 cm for all three axes.

**Tremor frequency and amplitude**

Tremor was introduced as an oscillatory disturbance affecting hand movements along the X-axis with the following characteristics:

- Maximum amplitude: 10 cm (in virtual space).
- Peak-to-peak displacement: 20 cm.
- Frequency: 2 Hz.

The tremor effect was included in trials based on the estimated difficulty level and remained a constant oscillatory disturbance, affecting movement precision similarly to physiological tremor patterns.

### Appendix B: CONSORT 2010 flow diagram

See Fig. 5.

### Appendix C: Age distribution by intervention group

See Fig. 6.

### Appendix D: Descriptive statistics of SSQ scores stratified by sex

See Table 5.

**Table 5:** Median and range of Simulator Sickness Questionnaire (SSQ) scores for female and male participants

	Total score (Median [Min–Max])	Nausea (Median [Min–Max])	Oculomotor (Median [Min–Max])	Disorientation (Median [Min–Max])
Female	13.1 [0–78]	9.5 [0–57]	15.2 [0–90]	13.9 [0–83]
Male	7.5 [0–48]	0.0 [0–38]	7.6 [0–53]	0.0 [0–83]

*n* = 62 female, *n* = 42 male participants

### Appendix E: User experience questionnaire responses

See Fig. 7.
